# Assessing the choice of National Health Insurance Fund contracted outpatient facilities in Kenya: A qualitative study

**DOI:** 10.1002/hpm.3693

**Published:** 2023-07-22

**Authors:** Jacob Kazungu, Justice Nonvignon, Matthew Quaife, Edwine Barasa

**Affiliations:** ^1^ Health Economics Research Unit KEMRI Wellcome Trust Research Programme Nairobi Kenya; ^2^ Department of Health Policy, Planning and Management School of Public Health University of Ghana Accra Ghana; ^3^ Health Economics Programme Africa Centres for Disease Control and Prevention Addis Ababa Ethiopia; ^4^ Department of Infectious Disease Epidemiology London School of Hygiene and Tropical Medicine London UK; ^5^ Nuffield Department of Medicine University of Oxford Oxford UK

**Keywords:** facility choice, Kenya, National Health Insurance Fund

## Abstract

**Objective:**

To assess National Health Insurance Fund (NHIF) members' level of understanding, experiences, and factors influencing their choice of NHIF‐contracted outpatient facilities in Kenya.

**Methods:**

We conducted a cross‐sectional qualitative study with NHIF members in two purposefully selected counties (Nyeri and Makueni counties) in Kenya. We collected data through 15 focus group discussions with NHIF members. Data were analysed using a framework analysis approach.

**Results:**

Urban‐based NHIF members had a good understanding of the NHIF‐contracted outpatient facility selection process and the approaches for choosing and changing providers, unlike their rural counterparts. While NHIF members were required to choose a provider before accessing care, the number of available alternative facilities was perceived to be inadequate. Finally, NHIF members identified seven factors they considered important when choosing an NHIF‐contracted outpatient provider. Of these factors, the availability of drugs, distance from the household to the facility and waiting time at the facility until consultation were considered the most important.

**Conclusion:**

There is a need for the NHIF to prioritise awareness‐raising approaches tailored to rural settings. Further, there is a need for the NHIF to contract more providers to both spur competition among providers and provide alternatives for members to choose from. Besides, NHIF members revealed the important factors they consider when selecting outpatient facilities. Consequently, NHIF should leverage the preferred factors when contracting healthcare providers. Similarly, healthcare providers should enhance the availability of drugs, reduce waiting times whilst improving their staff's attitudes which would improve user satisfaction and the quality of care provided.

## Highlights

- Patient choice of health facilities can enhance the attainment of health system objectives.
- Availability of drugs, distance and waiting time are the important factors influencing National Health Insurance Fund (NHIF) members' choice of outpatient facilities.
- NHIF should incorporate these preferences when accrediting and contracting providers
- Awareness‐raising communication should be tailored to rural setting needs.

## 1 BACKGROUND

Since the adoption of the resolution by the World Health Organisation (WHO) member states in 2005 to transform their health financing system towards achieving universal health coverage (UHC) by 2030, countries have been making reforms to accelerate progress towards meeting that target.^1^, ^2^, ^3^ Several studies including a 2010 WHO Report have re‐emphasised the role of health financing as a central area to leverage reforms for UHC.^4^, ^5^, ^6^, ^7^ While health financing reforms should ideally focus on all three health financing functions—revenue generation, pooling and purchasing—increasing evidence indicate that the purchasing function is often not given sufficient research emphasis.^8^

Kenya has prioritised purchasing reforms in her quest to achieve UHC^9^ and has implemented several reforms through the NHIF. The NHIF is the main public purchaser in Kenya that has been chosen as the ‘vehicle’ to drive the UHC agenda in Kenya. It establishes service entitlements for the beneficiaries, selects and contracts providers and reimburses them for services given on behalf of the beneficiaries.^10^ Several reforms have been implemented at the NHIF in a move to transform it into a strategic purchaser. For instance, prior to 2015, the NHIF only covered inpatient services, however, following reforms to the NHIF, it included outpatient cover where providers would be contracted and paid using capitation.^11^, ^12^, ^13^

Two key design features of the outpatient cover were (1) to selectively contract facilities and capitate NHIF members to those facilities, and (2) to allow NHIF members to choose outpatient facilities of their choice with an opportunity to change a facility once every quarter (3 months). The inclusion of these features was intended to not only drive down the costs of care as shown in the literature from other settings^14^ but also encourage competition among providers which has been shown to enhance efficiency, quality of care, equity, and access to healthcare services.^15^, ^16^, ^17^

The NHIF covers 24% of the population^18^ and members are required to voluntarily select outpatient facilities of their choice by either visiting an NHIF branch office, using the *My NHIF App* or using the Unstructured Supplementary Service Data (USSD) code *155# before accessing outpatient care. Evidence from other settings on patient choice have highlighted several factors that influence patient choice including waiting time to appointment or waiting time in practice, opening hours, price of care, distance to facility among other factors related to the structure process and outcome of care.^19^, ^20^, ^21^, ^22^

While allowing patient choice was a key step for NHIF to implement key strategic purchasing actions related to both providers and citizens,^23^ no study has examined how members perceived the available choices, their level of awareness of the process of choosing/changing providers and the factors they value before choosing a facility.

Against this backdrop, this study aimed to assess the NHIF members' level of understanding and experiences with the NHIF‐contracted outpatient facility selection process as well as the factors that influence their choice of outpatient facilities in Kenya. Findings from this study are crucial given the ongoing reforms to transform the NHIF into a strategic purchaser in Kenya and the dearth of patient choice studies especially in low‐ and middle‐income countries.

## 2 METHODS

### 2.1 Study setting

Kenya is a lower‐middle‐income country in East Africa with a decentralised form of government comprised of a national government and 47 county governments. The health system in Kenya is organised around four tiers made up of six levels of care: Tier 1—is comprised of community units; Tier 2—this is made up of primary care facilities comprised of dispensaries, clinics, and health centres; Tier 3—these are the county hospitals made, and Tier 4—comprising of the national referral hospitals.

Health is one of the devolved functions in Kenya. Inter alia, the national government is tasked with developing health policy and managing national referral hospitals (Tier 4/Level 6 hospitals) whereas county governments own and run lower‐level public facilities (Tiers 1–3). To implement the UHC programme in Kenya, the National government piloted the UHC programme across four counties (Kisumu, Nyeri, Isiolo and Machakos). During the implementation of the UHC programme in Kenya, several other counties including Makueni were also implementing their own county led UHC programmes. While the implementation of the UHC pilot was through input‐based financing—where counties/facilities received commodities/inputs and the whole population in those counties could access services for free, the scale‐up of the UHC programme in Kenya was targeted to indigents who were identified across counties and registered through the NHIF.

We conducted this study in two purposefully selected counties in Kenya, Nyeri and Makueni. The counties were included in this study to represent a county which had piloted the national‐level UHC programme (Nyeri) and a county (Makueni) that had both not implemented the UHC pilot but also had a locally‐run UHC programme. The use of the UHC programme implementation criteria in the inclusion of the study counties was to provide useful information to support the UHC scale‐up in Kenya and particularly through the NHIF. Table 1 highlights the demographic and selected health indicators of the two selected study counties.

**TABLE 1:** Distribution of demographic and selected health financing indicators in Kenya and the two selected study counties.

Indicator	Nyeri county	Makueni county	Kenya
Poverty rate (2015/16 KIHBS)^24^	19.3%	34.8%	36.1%
Population (2019 census)^25^			
Total	759,164	987,653	47,564,296
Male	374,288	489,691	23,548,056
Female	384,845	497,942	24,014,716
Intersex	31	20	1524
Number of health facilities^26^			
Total	443	352	14,032
Public	143	242	6451
Private	272	83	6549
Faith‐based	28	27	1032
Health financing			
CGHE as a % of TCGE in 2018/19^27^	22.2%	55.5%	11.7%
Per capita health spending in KES in 2018/19^27^	3326	2882	10,703
Health insurance coverage in 2014^28^			
Total	31.4%	12.3%	19.8%
NHIF	27.1%	10.6%	15.9%
Private	1.9%	0.5%	1.2%

Abbreviations: CGHE, County General Health Expenditure; KIHBS, Kenya Integrated Household Budget Survey; TCGE, Total County Government Expenditure.

### 2.2 Study design and data collection

We adopted a qualitative cross‐sectional approach where we engaged NHIF members through focus group discussions (FGDs) across the two counties. Focus group discussions were preferred as opposed to other approaches such as in‐depth interviews to help us gain insights into the general perspectives^29^ of NHIF members' choices around facility selection and to reach many members over a shorter period. Besides, the FGDs were more economical to use in this research as opposed to in‐depth interviews—Schwab et al describes the pros and cons of the two approaches.^30^

We collected data from registered NHIF members who had NHIF membership cards. Initially, in the first county, we obtained a list of all registered NHIF members and made phone calls to invite them for FGDs. However, this approach failed, as (1) most of the numbers could not go through, for instance, because members had changed phone numbers thus reaching different people, and (2) people were not living within the county during the study period. Consequently, we resorted to using community health volunteers (CHVs) to mobilise NHIF members from rural and urban areas. The CHVs reached out to households within their community through door‐to‐door and invited willing household members who were NHIF members (had an NHIF card) to participate in the FGDs on a selected date and venue. The researcher (JK) and the CHVs utilised the maximum variation sampling approach to purposefully include gender variation and age distribution of participants included in each of the FGDs.

**FIGURE 1:**
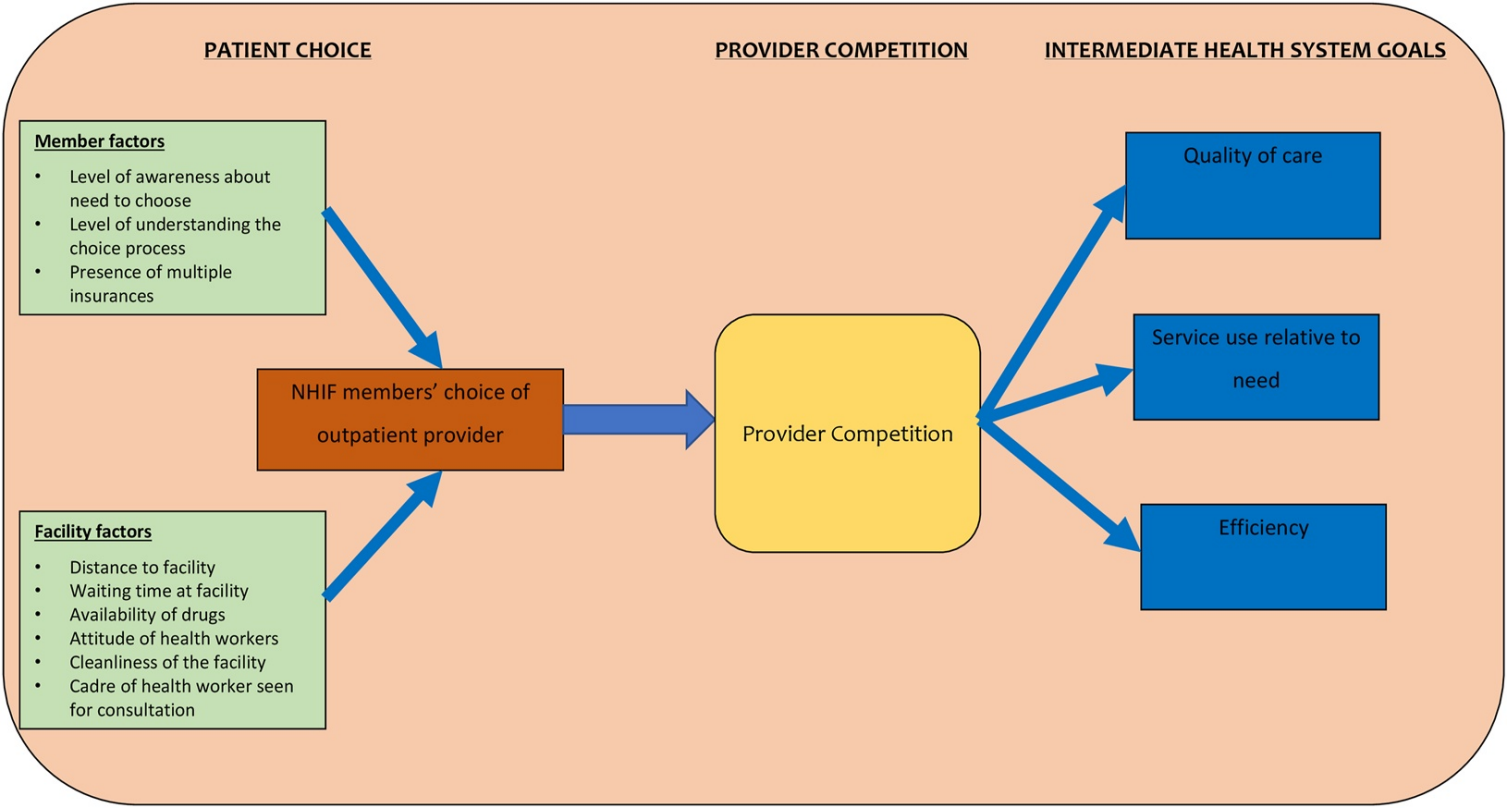
Conceptual framework.

The FGDs were conducted using a semi‐structured topic guide which was developed around factors conceptualised to affect the choice of an outpatient facility among NHIF members. Evidence from other settings had shown that patients' or a population's choice of health facilities is often influenced by the cost of care,^31^, ^32^, ^33^, ^34^ availability of medical equipment and drugs,^35^, ^36^, ^37^ distance to the facility,^35^, ^38^, ^39^ the quality of services provided,^31^, ^39^, ^40^, ^41^ waiting time and consultation time,^31^, ^36^, ^40^ and the staff attitudes.^33^, ^37^, ^39^ These were categorised as individual and facility‐level factors (Figure 1). We collected data from a total of 148 NHIF members through 15 FGDs—each taking between 40 and 90 min. Out of the 15 FGDs, eight were conducted in Makueni county (4 in rural and 4 in urban areas) while 7 were conducted in Nyeri county (4 in urban and 3 in rural areas). Out of the 148 NHIF members, 96 were women and 52 were men. The average age of the participants was 52.6 years but ranged from 23 to 74 years.

The FGDs were conducted in English and Swahili and audio recorded. All FGDs were conducted in a central area selected by the participants in both rural and urban centres and were facilitated by JK. Data collection stopped when data saturation was achieved. Data collection was conducted between July and December 2021.

### 2.3 Conceptual framework

We conceptualised the study framework based on Dixon's et al. (2010) analytical framework of patient choice and Kutzin's et al. (2013) health system goals.^5^, ^15^

Our framework identifies individual and health facility‐level factors that influence an NHIF member's choice of an outpatient healthcare provider. Given the choice that NHIF members have to make for outpatient facilities, it is postulated that the choice would create competition among providers to both be selected and retained by NHIF members. Consequently, provider competition drives health system service improvements especially the quality of care, efficiency and service use relative to need.^15^

### 2.4 Data analysis

Audio recordings from the FGDs were first transcribed verbatim in English. Audios conducted in Swahili were first transcribed verbatim in Swahili then translated into English. Data were analysed following a framework analysis approach following four key steps^42^, ^43^: (1) an initial reading through the transcripts to familiarise while identifying important factors that influence NHIF members' choice of an outpatient provider, (2) coming up with a coding framework, (3) a thorough second reading of the transcripts and matching identified contents from each transcript onto the coding framework, and (4) recording the matched data and analysing across key areas related to NHIF members' level of awareness and factors influencing the choice of an NHIF‐contracted outpatient provider. The coding process was conducted manually in Microsoft (MS) Excel.

## 3 RESULTS

### 3.1 NHIF members' level of awareness of and experiences with the outpatient facility selection and change process

Overall, NHIF members had a good understanding of outpatient facility selection/change and the process involved, however, there were notable variations in the level of awareness for members from rural versus urban areas. For instance, while all NHIF members from urban areas were aware of the requirement to select outpatient facilities prior to receiving services, NHIF members from rural areas struggled to describe the process of outpatient facility selection and were largely not aware of the requirement to choose a facility prior to accessing services.

Further, NHIF members from urban areas were also very conversant with the mode of changing facilities compared to their counterparts engaged in rural settings. The awareness about the selection and change of facility process was largely factored by the availability of NHIF branch offices closer to members from urban areas which enabled them to access information by walking in to have their questions answered compared to respondents in rural settings.

I have been made aware that it is important to choose an outpatient facility where I can be treated without being admitted… you are told to choose a hospital where you can get to fast in case there is an emergency. If there is a hospital which is near you and is offering NHIF services and maybe you do not like it, you will just have to choose it. FGD 4 Nyeri County ‐ Urban

I did not choose, but I just used it [accessed NHIF services], I became ill and I had to go to a nearby hospital, So, when I went there, they chose for me themselves. FGD 3 Makueni County – Rural

With regards to awareness about the approaches used to select an outpatient provider, respondents indicated that several methods are used to select or change a facility. First, respondents indicated that an NHIF member can visit the NHIF branch to choose a facility. Second, NHIF members also indicated that they could use the ‘*My NHIF*’ application or website to select or change a facility of their choice. Lastly, the respondents also indicated the use of the USSD code ‘*155#’ on their mobile phones to choose or change a facility. Generally, respondents highlighted the ease of using the mobile‐based approaches to selecting a facility and the fact that one does not require to visit the NHIF branch office to choose or change a facility.

Selection was easy because the process has been made easier, through this application. It is not the era whereby you have to move to the NHIF offices and start queuing and maybe follow up with a long queue, but now it is easy because it’s just a click of a button. If it’s an application you just click, if it’s *155# It's very easy so the selection is not an issue. FGD 1 Makueni County ‐ Urban.

There was a time I chose private but you know these days there is that plan of changing after three months and you choose what you need, so I have changed and chosen the Government FGD 2 Makueni County – Rural

Although some participants in rural areas were aware of this process, a majority were surprised that such easier platforms existed and could be used to select or change facilities. This group that was not aware, however, had either not selected a facility or had their facilities selected when the facility selection process was done at facilities themselves rather than via online or USSD code approach.

The key challenge highlighted was the fact that airtime was required for one to use the *155# approach and data bundles are required to use the App. Respondents would prefer the USSD code approach to be free of charge.

I did not choose because I did not know about that * 155 # but I just wanted to get to the nearest facility to receive treatment FGD 3 Makueni county – Rural

However, respondents also highlighted the restriction of choice given the number and facility levels that were contracted by NHIF to provide outpatient services. It was pointed out that, most of the lower‐level facilities which were closer to the respondents especially in rural settings were not within the list of contracted facilities and therefore NHIF members had to choose contracted facilities that were often far.

On the other hand, some respondents highlighted that, although they chose a facility, they did not like the services provided but due to the extra costs to be incurred travelling to an alternative facility, they had to remain at that facility as it was closer.

I was told that I can’t select a dispensary because a dispensary cannot take NHIF. There are hospitals which you are given to choose from which have NHIF FGD 3 Nyeri County ‐ Rural

For outpatient, maybe you are facing a challenge with the fare on and off, going and coming back, you will be forced to choose a hospital in Emali since it is the one closest to your home and maybe you are not satisfied with the services. FGD 4 Makueni County ‐ Rural

### 3.2 Reasons for not selecting an NHIF‐contracted outpatient facility

Several reasons were highlighted for those that had not yet selected an outpatient facility. First, these members, particularly those from rural settings, expressed a lack of awareness about the requirement for them to choose a facility before accessing services. Second, this was further exacerbated by the fact that a majority of members from rural areas were not very conversant with the technological approaches for choosing a facility other than visiting an NHIF branch or having their facility chosen for them when they visited a facility while ill. Third, while NHIF was giving members a choice to choose a facility, members expressed the lack of choice especially in rural areas where only one or very few NHIF‐contracted facilities existed, which were often very far. Finally, other members especially in urban settings had other insurance mechanisms, particularly, private insurance which they used mostly.

There is that challenge about outpatient, you get other people are not literate and now if you tell him to dial *155#, it becomes a challenge FGD 4 Nyeri County – Rural

I did not choose because I did not know about that * 155 # but I just wanted to get to the nearest facility to receive treatment FGD 1 Makueni County – Rural

My employer also provides me with a private health insurance that covers me and my family, I don’t see the need to use NHIF despite contributing every month….so I have not yet selected an outpatient provider FGD 4 Nyeri County ‐ Urban

### 3.3 Factors that influence NHIF members' choice of an outpatient provider

#### 3.3.1 Availability of drugs

National Health Insurance Fund members stated that they would select facilities where drugs were available. Drugs were considered to be the bare minimum criterion given the price of obtaining the drugs in private pharmacies and the fact that often people could fall sick when they don't have money to buy drugs out‐of‐pocket.

It is better you use ten shillings to go and ten shillings to come back and you get drugs, so [I am] saying, availability of drugs is a very important issue. FGD 4 Nyeri County – Rural

Drugs are a must. You can go there and get a prescription and yet you only had bus fare. When you go home without drugs, you will continue to be sick. FGD 3 Nyeri County – Urban

#### 3.3.2 Distance from the household to the facility

National Health Insurance Fund members highlighted distance to be an important factor influencing their choice of an outpatient provider. Importantly, NHIF members indicated a preference for facilities that were not longer than 5 KM from their households to reduce both the suffering in moving from a household to a facility when ill and the associated costs for travelling to a far facility. However, some members, especially in rural settings highlighted a willingness to travel to a far healthcare provider as long as they would obtain drugs.

Distance to the facility is also very important. Imagine you need urgent care and the facility is very far, you will be in trouble. It’s better a facility that is close, maybe three or five Kilometres. Again, the far the facility is, you have to pay for transport to get there FGD 4 Nyeri County – Rural

#### 3.3.3 Waiting time at the facility until consultation

Although the availability of drugs and distance to facilities were often ranked by NHIF members as the top two factors influencing their choice of an outpatient healthcare provider, NHIF members also indicated a high preference for the waiting time at the facility. Waiting time was defined as the time a patient would wait in line at the facility until they get a consultation. Notably, even though respondents indicated an acceptable waiting time of up to 3 h, respondents in rural areas were more likely to state longer waiting times even up to 5 hours than their counterparts in FGDs conducted in urban areas. A key reason for this was as a result of fewer NHIF‐contracted outpatient providers in rural areas than in urban settings where several NHIF‐contracted private and public facilities were contracted by NHIF and available for selection.

Sometimes you will find that the hospital is full to capacity, long queues so before you get out of there, you will have lost a lot of time, it will be a difficult time for you. FGD 1 Makueni County – Rural

#### 3.3.4 The attitude of the health workers

National Health Insurance Fund members indicated other factors associated with the quality of care received within facilities. The attitude (courteousness) of the healthcare worker was one of the highlighted factors. Respondents expressed issues around the level of respect accorded to them by healthcare workers some complaining about issues of abuses and the lack of proper explanation of required procedures. National Health Insurance Fund members expressed a preference for facilities where the staff would talk to them nicely and with respect as opposed to facilities where the staff were rude and used abusive language.

It is how the doctors receive you, how they talk to you, the nurse calls you as if you are [not human] that public relation should be very improved, public relation for these people. FGD 4 Makueni County – Urban

You can go there and see how they serve people, even how the nurses talk to patients, they are very harsh, and sometimes they irritate you even before you are treated. I look for a hospital which I have gone to before and I have seen them serve people well. FGD 4 Nyeri County – Rural

#### 3.3.5 The cadre of staff seen during consultation

The cadre of healthcare workers seen during the consultation was also an important factor. In urban settings particularly, NHIF members indicated the need to see a Medical Doctor/Officer rather than a clinical officer or nurse. Respondents expressed the need for a more experienced health worker cadre for consultation and therefore a more senior medical personnel would be ideal. However, in rural areas, even though this factor was also highlighted, it was not a major contributor to the choice.

I think medical doctors are the most experienced staff and would treat me better than seeing a nurse. FGD 4 Makueni county – Urban

I think all staff that are at the facility are qualified enough to provide care at that level. I wouldn’t mind seeing a nurse or clinical officer or medical doctor as long as they will treat me well and I would get the medication I need. I actually can’t tell the difference between a clinical officer and a medical doctor! FGD 4 Nyeri County – Rural

#### 3.3.6 Cleanliness of the facility

The cleanliness of a facility was another quality‐related factor that influenced the choice of an outpatient facility among NHIF members. Cleanliness was defined around the cleanliness of the consultation rooms, facility floors and toilets. Members preferred facilities that were always clean especially the corridors, waiting areas, consultation rooms and hygiene facilities. Cleanliness of a facility was indicated to be important due to general view or hygiene reasons as well as the likelihood to get other infections, especially where a facility was not tidy enough.

Even though there are other very important factors but the facility should be clean. Cleanliness is very important as it shows how the facility provides quality services or not. FGD 1 Makueni County – Rural

#### 3.3.7 Opening hours of the facility

As outpatient services do not involve admission, the opening hours of the facility was another important factor. Specifically, NHIF members highlighted a preference for facilities that were open 24 h a day compared to those only open during the day. This was crucial as people could require care at any time even at night.

I would choose a facility that is open 24 hours a day compared to one that is 12 hours a day. I can have a very bad headache at night and need urgent medical attention or drugs. It would be very bad if the facility is closed at night. FGD 1 Makueni County – Rural

## 4 DISCUSSION

Our study presents the level of awareness, experiences and factors that influence NHIF members' choice of an outpatient facility in Kenya. We show that, first, while there is a good understanding of the NHIF requirement to choose a facility, members from rural areas remain largely unaware of the electronic approaches to choosing or changing a facility.

While technology has been shown to improve the ease of choosing and changing facilities in this study, it appears the approach to communicating the changes, especially in a rural setting remains wanting. While methods such as the media and the Internet are mostly used by NHIF to pass information, the rural setting population often don't have good access to media or the Internet and thus such information may not reach them. For instance, as of 2020, only 30% of the population in Kenya was using the Internet.^44^ Besides, a larger share of the population in Kenya is either not exposed to media or exposed less than once a week^28^ making obtaining information passed by NHIF through such platforms a key challenge. Besides, these findings align with a previous study where respondents expressed the inadequate communication of the new benefits package introduced by NHIF and even when communication was done, it was unequally distributed across different citizen groups.^11^

Second, respondents also expressed concern regarding the lack of choice for providers in rural settings due to the few facilities contracted to offer outpatient services. These findings are similar to those from two previous studies in Kenya that showed that NHIF contracting of facilities has had an urban bias and the contracting process undermined equity.^11^, ^45^ While the contracting process involves an application for accreditation, inspection, gazettement and contract signing, the process has been shown to undermine geographical access, especially in rural areas and historically marginalised settings in Kenya due to the rigorous nature of the requirements that leave out the only available facilities that do not meet the conditions in the marginalised areas.^45^ Besides, while the process is initiated by the NHIF in the public sector, private providers self‐initiate the process of contracting which may explain the fewer providers from the private sector even though the sector forms over 50% of all providers in Kenya.^26^ Furthermore, providers have expressed dissatisfaction with the provider payment rates and mechanisms used by NHIF^46^ which could further explain the fewer providers willing to be contracted by NHIF.

Third, it is not surprising that the availability of drugs, distance from the household to the facility and waiting time at the facility were the three most important factors influencing NHIF members' choice of an NHIF‐contracted outpatient provider. These findings are similar to those reported in other studies.^19^, ^35^, ^36^, ^37^, ^38^, ^39^ These can be explained. First, the availability of drugs in a facility was considered the most important factor perhaps due to the fact that medicines often account for the largest share of costs for accessing care. For instance, a study among diabetes patients in Kenya showed that medicines alone accounted for 52.4% of the average annual direct patient costs in Kenya.^47^ Besides, 36.1% of the population in Kenya is poor making the purchase of medicines an additional burden thus the preference for facilities that they know they would get medicines.^24^

Distance from the household to a health facility was the second most important factor influencing NHIF members' choice of an outpatient provider. Similar to medication, distance to facilities places both a financial and physical burden on NHIF members as it influences both the transport costs to a facility and the suffering as one moves from their household to the facility. Transport costs were the second significant contributor to direct patient diabetes costs among patients in Kenya.^47^ In another study in Kenya, transport costs alone increased the incidence of catastrophic health expenditure from 4.52% to 6.58% with a larger share of this being in rural settings where individuals from rural settings were over five times more likely to experience catastrophic health expenditure from transport costs to health facilities compared to their urban counterparts.^48^ Even though the government targets to have the whole population to be within a 1‐h travel time to a health facility, spatial access estimates indicate that nearly 11% of the population still leaves outside the 1‐h travel time to facilities.^49^ Anecdotal evidence suggests that spatial access estimates may be worse for NHIF‐contracted outpatient facilities given that NHIF has contracted less than half of the facilities in Kenya.

It is not surprising that waiting time at the facility until consultation was the third most important factor that NHIF members considered important. This finding is similar to what has been reported in other studies.^31^, ^36^, ^40^ For instance, in South Africa, Honda et al. found that waiting time was also an important non‐clinical quality of care factor that influenced attendance to public health facilities.^36^

## 5 LIMITATION

Findings from our study should be interpreted in light of the following limitation. The findings from the study may not be generalisable to the whole country given the approach and number of counties included. Despite this, these findings provide nuances to the literature on people or patients' choice of health facilities and provide evidence to inform further NHIF reforms for UHC in Kenya.

## 6 POLICY IMPLICATIONS

This limitation notwithstanding, the study offers several policy implications. First, for communication between NHIF and NHIF members especially in rural settings to be effective, there is a need to use locally relevant platforms that members can easily interact with such as local radio stations rather than via the website or social media platforms that are not accessible. Evidence in the health sector have shown a higher preference and uptake of communication when community radio is used.^50^, ^51^, ^52^ Second, NHIF should revise the contractual process with keen consideration on revising provider payment mechanisms and how facilities from historically marginalised areas are inspected. Third, the NHIF should initiate the process of contracting private providers, especially in areas where few public providers exist and where alternative providers do not exist within the 1‐h travel time from the population's households. Fourth, the government and private facilities should prioritise the availability of drugs in their facilities to make sure that the population can access them rather than have to buy elsewhere out‐of‐pocket. Related to this, the NHIF should ensure that contracted facilities have drugs every quarter perhaps through close supervision. Fifth, providers should also prioritise the other important factors highlighted, particularly, reducing waiting times at the facility, improving the attitudes of their staff, enhance the cleanliness of the facility and the opening hours with a focus to have the facility open day and night including weekends.

## 7 CONCLUSION

Our study highlighted an urban‐biased level of awareness of the NHIF‐contracted outpatient facility selection process among NHIF members in Kenya and the important factors that NHIF members consider when choosing a facility. Therefore, there is a need to address barriers that limit awareness reach among rural NHIF members whilst incorporating factors preferred by NHIF members into contracting arrangements between NHIF and healthcare providers as well as service provision improvement at the facility level.

## AUTHOR CONTRIBUTION

JK conceptualised the study. All authors contributed to the development of the FGD guide. JK collected the data. JK developed the coding frame and reviewed by all other authors. JK drafted the initial manuscript and reviewed by all authors. All authors read and approved the final manuscript.

## CONFLICT OF INTEREST STATEMENT

The authors declare no conflict of interest.

## ETHICS STATEMENT

Ethical approval for the study was obtained from the Scientific Ethics Review Unit (SERU) of KEMRI (Ref: KEMRI/SERU/CGMR‐C/191/4019) and the County Departments of Health in both study counties. Also, we obtained permission to conduct the study from the NHIF, the National Commission for Science, Technology and Innovation (NACOSTI) and the Council of Governors in Kenya.

## CONSENT TO PARTICIPATE

We also obtained written consent from each participant prior to conducting the FGDs.

## Data Availability

The data that support the findings of this study are available on request from the corresponding author. The data are not publicly available due to privacy or ethical restrictions.
